# Supplementary material for: Implementation of Newborn Screening for Conditions in the United States First Recommended during 2010–2018
Source: Int J Neonatal Screen. 2023 Apr 6;9(2):20. doi: 10.3390/ijns9020020 (PMC10123615; doi:10.3390/ijns9020020)
Supplement: Supplementary file 1 [file IJNS-09-00020-s001.zip › IJNS-2220364-Supplementary.pdf]

Supplementary Materials:

Table S1: Years from Addition to RUSP to Full Population Screening (as of December 2022)						
**Condition (Month/Year Added to RUSP)**	**SCID (May 2010)**	**CCHD (September 2011)**	**Pompe (March 2015)**	**MPS I (February 2016)**	**X-ALD (February 2016)**	**SMA (July 2018)**
**AVERAGE**	4.3	2.7	4.0*	3.2*	3.5*	2.1*
**Alabama**	8.3	1.8	PI	PI	PI	3.6
**Alaska**	6.2	2.3	NS	NS	NS	4.0
**Arizona**	7.3	3.8	PI	PI	4.9	3.5
**Arkansas**	5.0	1.9	NS	NS	6.0	1.7
**California**	0.3	1.8	3.4	2.5	0.6	1.9
**Colorado**	1.8	4.3	7.3	6.3	NS	1.5
**Connecticut**	1.3	1.3	5.8	4.9	0.4	1.5
**Delaware**	2.2	1.7	4.8	3.9	3.9	1.5
**District of Columbia**	3.8	4.0	3.1	2.2	2.2	PI
**Florida**	2.1	3.1	4.9	4.0	2.3	1.8
**Georgia**	6.1	2.8	6.5	5.6	4.3	1.1
**Guam**	3.9	1.8	NS	NS	NS	NS
**Hawaii**	4.8	3.8	PI	PI	NS	NS
**Idaho**	5.7	6.8	6.9	6.0	6.0	3.6
**Illinois**	4.1	2.0	0.3	-0.7	3.3	1.9
**Indiana**	8.2	0.3	5.3	4.4	5.4	0.0
**Iowa**	4.2	1.8	PI	PI	NS	3.2
**Kansas**	7.5	6.4	5.8	4.9	PI	1.6
**Kentucky**	5.8	2.3	0.9	0.0	2.4	1.1
**Louisiana**	8.6	1.9	6.8	5.9	NS	3.5
**Maine**	4.3	1.8	6.1	5.2	5.2	2.8
**Maryland**	5.8	2.0	4.3	3.3	PI	0.9
**Massachusetts**	-1.2	3.3	2.8	1.9	1.9	-0.5
**Michigan**	1.3	2.6	2.4	1.5	3.7	1.7
**Minnesota**	2.7	1.9	2.4	1.5	1.0	-0.3
**Mississippi**	1.6	3.2	1.3	NS	NS	1.3
**Missouri**	6.7	2.3	-2.2	-3.1	5.8	0.5
**Montana**	5.2	2.8	PI	PI	NS	2.7
**Nebraska**	4.3	2.0	3.3	2.4	2.4	2.3
**Nevada**	7.7	3.8	NS	NS	NS	NS
**New Hampshire**	5.2	0.9	5.4	4.5	4.5	1.4
**New Jersey**	4.1	0.0	4.3	3.4	6.8	3.5
**New Mexico**	4.3	2.8	6.2	5.3	PI	3.9
**New York**	0.3	2.3	-0.4	2.7	-2.9	0.3
**North Carolina**	7.5	2.8	PI	PI	6.0	2.8
**North Dakota**	6.2	1.9	PI	NS	NS	3.2
**Ohio**	3.2	3.1	2.7	1.8	6.7	4.3
**Oklahoma**	4.8	1.8	6.0	5.1	5.1	2.7
**Oregon**	4.0	1.8	3.6	2.7	NS	3.9
**Pennsylvania**	3.2	3.0	0.9	0.9	1.2	0.7
**Puerto Rico**	5.2	3.2	NS	NS	NS	NS
**Rhode Island**	4.3	3.8	2.8	1.9	1.9	2.0
**South Carolina**	4.8	1.8	5.9	5.0	PI	4.2
**South Dakota**	5.3	1.8	7.5	PI	PI	3.2
**Tennessee**	5.7	1.7	2.3	1.4	2.3	1.6
**Texas**	2.6	2.0	PI	PI	3.5	2.9
**Utah**	3.2	3.1	PI	PI	4.6	-0.5
**Vermont**	6.2	4.8	4.2	3.3	3.3	0.8
**Virginia**	5.1	3.3	3.8	2.9	6.1	3.7
**Washington**	3.7	3.8	4.6	3.7	2.1	2.1
**West Virginia**	3.8	0.8	NS	NS	PI	1.3
**Wisconsin**	-1.8	2.5	6.8	NS	PI	1.3
**Wyoming**	1.8	6.3	NS	NS	NS	1.5
**MINIMUM**	-1.8	0	-2.2	-3.1	-2.9	-0.5
**MAXIMUM**	8.6	6.8	7.5*	6.3*	6.8*	4.3*

*Of states offering Universal Screening as of December 2022 | NS = Not Screened | PI = Pursuing Implementation

Table S2: Number of states that implemented population screening within 3 years of a conditions' addition to the RUSP		
**Condition**	**Number of States**	**States**
SCID	13	California, Colorado, Connecticut, Delaware, Florida, Massachusetts, Michigan, Minnesota, Mississippi, New York, Texas, Wisconsin, Wyoming
CCHD	35	Alabama, Alaska, Arkansas, California, Connecticut, Delaware, Georgia, Guam, Illinois, Indiana, Iowa, Kentucky, Louisiana, Maine, Maryland, Michigan, Minnesota, Missouri, Montana, Nebraska, New Hampshire, New Jersey, New Mexico, New York, North Carolina, North Dakota, Oklahoma, Oregon, Pennsylvania, South Carolina, South Dakota, Tennessee, Texas, West Virginia, Wisconsin
Pompe	12	Illinois, Kentucky, Massachusetts, Michigan, Minnesota, Mississippi, Missouri, New York, Ohio, Pennsylvania, Rhode Island, Tennessee
MPS I	16	California, District of Columbia, Illinois, Kentucky, Massachusetts, Michigan, Minnesota, Missouri, Nebraska, New York, Ohio, Oregon, Pennsylvania, Rhode Island, Tennessee, Virginia
X-ALD	13	California, Connecticut, District of Columbia, Florida, Kentucky, Massachusetts, Minnesota, Nebraska, New York, Pennsylvania, Rhode Island, Tennessee, Washington
SMA	34	Arkansas, California, Colorado, Connecticut, Delaware, Florida, Georgia, Illinois, Indiana, Kansas, Kentucky, Maine, Maryland, Massachusetts, Michigan, Minnesota, Mississippi, Missouri, Montana, Nebraska, New Hampshire, New York, North Carolina, Oklahoma, Pennsylvania, Rhode Island, Tennessee, Texas, Utah, Vermont, Washington, West Virginia, Wisconsin, Wyoming

Table S3: Severe Combined Immunodeficiency Newborn Screening Implementation Dates (N = 53)

						NM				
						NE				
						RI				
						ME	SD			
						IA	PR	VT		
						NJ	NH	ND		
				TX		IL	MT	AK		
				DE		OR	VA	GA		
				FL	UT	GU	AR	MD	NC	LA
				WY	PA	WV	SC	KY	KS	AL
		NY	MI	CO	OH	DC	HI	TN	AZ	IN
WI	MA	CA	CT	MS	MN	WA	OK	ID	MO	NV
**2008**	**2009**	**2010 RUSP**	**2011**	**2012**	**2013**	**2014**	**2015**	**2016**	**2017**	**2018**

Table S4: Critical Congenital Heart Disease Newborn Screening Implementation Dates (N = 53)

TX	
NE	
MD	
IL	VA

		ND	PR				
		MN	MS				
		LA	UT				
		AR	OH				
		SD	FL				
		OK	PA				
		ME	NC				
		IA	NM				
		CA	MT				
		SC	GA	DC			
		OR	MI	WA			
		GU	WI	RI			
		AL	NY	NV			
	NH	TN	MO	HI			
	WV	DE	KY	AZ	VT		KS
NJ	IN	CT	AK	MA	CO	WY	ID
**2011**	**2012**	**2013**	**2014**	**2015**	**2016**	**2017**	**2018**
**RUSP**							

Table S5: Pompe Newborn Screening Implementation Dates (as of December 2022; n = 37)

								GA	
								NM	
					OR			ME	
					CA	WA		OK	SD
				OH	NE	NJ		SC	CO
			MS	MN	DC	VT	NH	KS	ID
			PA	MI	RI	VA	IN	FL	WI
MO	NY	IL	KY	TN	MA	MN	DE	CT	LA
**2013**	**2014**	**2015**	**2016**	**2017**	**2018**	**2019**	**2020**	**2021**	**2022**
		**RUSP**							

Table S6: Mucopolysaccharidosis, Type I Newborn Screening Implementation Dates (as of December 2022, n = 34)

								GA	
					NY			NM	
					OR			ME	
				OH	CA	WA		OK	
				MN	NE	NJ		SC	
				MI	DC	MD	NH	KS	CO
				TN	RI	VT	IN	FL	ID
MO		IL	KY	PA	MA	VA	DE	CT	LA
**2013**	**2014**	**2015**	**2016**	**2017**	**2018**	**2019**	**2020**	**2021**	**2022**
			**RUSP**						

Table S7: X-linked Adrenoleukodystrophy Newborn Screening Implementation Dates (as of December 2022, n = 30)

					NE				
					KY				
					TN				NJ
					FL			MO	AR
					DC	MI	UT	IN	OH

					WA	TX	NH	ME	VA
			CA	PA	RI	IL	GA	OK	NC
NY			CT	MN	MA	VT	DE	AZ	ID
**2013**	**2014**	**2015**	**2016 RUSP**	**2017**	**2018**	**2019**	**2020**	**2021**	**2022**

Table S8: Spinal Muscular Atrophy Newborn Screening Implementation Dates (as of December 2022, n = 48)

		NE		
		WA		
		RI		
		IL		OH
	NH	CA		SC
	WV	FL		AK
	MS	MI	SD	OR
	WI	AR	ND	NM
	KY	TN	IA	VA
NY	GA	KS	TX	ID
IN	MD	WY	OK	AL
MN	VT	DE	NC	NJ
UT	PA	CT	ME	LA
MA	MO	CO	MT	AZ
**2018 RUSP**	**2019**	**2020**	**2021**	**2022**
